# Crimean-Congo hemorrhagic fever with hemophagocytic lymphohistiocytosis

**DOI:** 10.1590/0037-8682-0438-2020

**Published:** 2021-03-08

**Authors:** Nurhayat Yakut, Eda Kepenekli, Omer Dogru

**Affiliations:** 1 Marmara University School of Medicine, Division of Pediatric Infectious Diseases, Department of Pediatrics, Istanbul, Turkey.; 2 Marmara University School of Medicine, Division of Pediatric Hematology and Oncology, Department of Pediatrics, Istanbul, Turkey.

A previously healthy 14-year-old boy was admitted to the emergency room with complaints of fever, abdominal pain, headache, nausea, and vomiting for three days. Five days earlier, his mother had found two ticks on his back and abdomen. On admission, he appeared unwell, with a body temperature of 38.5°C. An abdominal examination revealed splenomegaly and diffuse tenderness on palpation. The initial laboratory testing revealed thrombocytopenia, leukopenia, and elevated liver enzymes. His fever, leukopenia, and thrombocytopenia persisted, with a platelet count of 10,000/mm^3^. Bone marrow aspiration was performed to rule out the possibility of malignancies. An increased ferritin level of 1,942 µg/L (7-140 µg/L) was observed. The bone marrow aspirate demonstrated hypocellularity and hemophagocytosis (Figure A and B), and all other data (fever, >38.5°C; splenomegaly; platelet count, 10,000/mm^3^; white blood cell count, 1,300/mm^3^; and, serum ferritin, 1,942 ng/mL) were consistent with the diagnostic criteria of hemophagocytic lymphohistiocytosis^1^ (HLH). Moreover, Crimean-Congo hemorrhagic fever (CCHF) viral RNA was detected by a polymerase chain reaction analysis. Crimean-Congo hemorrhagic fever is a tick-borne viral infection caused by the CCHF virus, a member of the Nairovirus group of the Bunyaviridae family. The patient was diagnosed with HLH attributable to CCHF and was successfully treated with ribavirin, intravenous immunoglobulin (IVIG), and supportive therapy. His leucopenia, thrombocytopenia, and elevated levels of liver enzymes, creatine phosphokinase enzymes, and ferritin improved after 48 h of IVIG administration. He was discharged without any sequelae. CCHF is one of the most severe viral zoonotic diseases in humans^2^ ^,^ ^3^, and HLH is one of its uncommon complications**.** Both CCHF and HLH have very high mortality rates in the absence of timely medical intervention. For this reason, a high index of suspicion should be maintained for both conditions, and the required examinations should be performed for the differential diagnosis. Although complications such as HLH are rare, they may increase the mortality of CCHF**.** We should be aware of this association to ensure rapid diagnosis and treatment of these diseases.

**FİGURE A:**
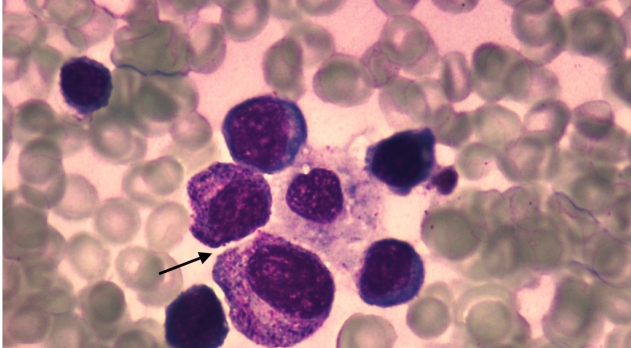
A bone marrow aspiration smear of hemophagocytosis (May-Grünwald-Giemsa (MGG) stain; original magnification, 1,000).

**FİGURE B:**
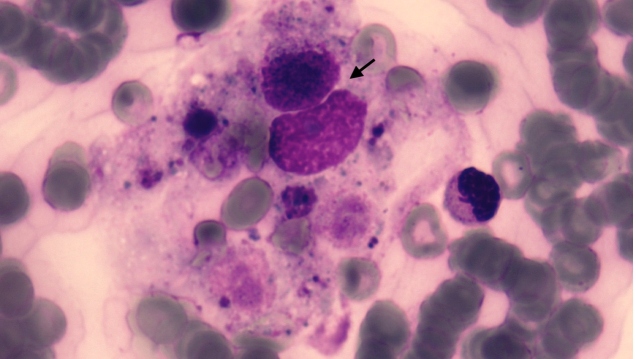
Bone marrow aspirate shows the phagocytosis of neutrophils and erythrocytes by the hemophagocyte (MGG stain stain; original magnification, 1,000).
